# The Late Dr. Ashbel Smith—Announcement

**Published:** 1886-02

**Authors:** 


### EDITORIAL NOTES.

IMPORTANT! THE LATE COL. (DR.) ASHBELL SMITH.—Although Dr. Smith had not been in active practice for many years, he was still regarded by many as the "Nestor" of Texas medicine. In consideration of the very general esteem in which he was held, we announce that *in our April number we will publish an excellent engraving of him* from a photograph taken when he was in excellent health, and the best one of him, extant; and at the same time, we will publish a *complete biography of the distinguished gentleman*, which Judge Golthwaite and Dr. D.F. Stewart, two of his most intimate friends and associates, have kindly consented to prepare.

The April number of the Journal will, therefore, be *very valuable* and much sought after, and as a limited number (only

500 in excess of our edition) will be published, those who have not already done so, should *subscribe now*, in order to secure a copy. It will pass into history; the picture we have, being the last Dr. Smith ever had taken, and is an exellent likeness —as he appeared in *his eightieth year*.

